# Supplementary material for: Chicken LEAP2 Level Substantially Changes with Feed Intake and May Be Regulated by CDX4 in Small Intestine
Source: Animals (Basel). 2022 Dec 11;12(24):3496. doi: 10.3390/ani12243496 (PMC9774203; doi:10.3390/ani12243496)
Supplement: Supplementary file 1 [file animals-12-03496-s001.zip › Figure S1.pdf]

```
                                                                   SP1 (-1000/-984)
-1050 AGTGCAAAAT AAAGACCGGT TTTGATTCCT CCTCACAGGG AAGCAAGACA AATAAGGGGA
      SPI1 (-999/-983)
 -990 AGTGGGAAAA TGTAAGAGGA ACCCCAGGAA AGGAACAAAT CTTCCAGCAG TGGGTTGGAA
 -930 GCAAAAGGGT TTTTGCTGCA AATTTGGGCT TCACAGTAAT CATCTGTAGG CTGAAACCTG
 -870 ACAACATAGC AGGCATTATC TTAATCTATG TAACTTGCCT TCCACGCTGG CACTTATGCC
 -810 TCCATATTAA GCAATCACAG AAAACTGTAG CCTATTGCAT TCACATCTGG ATTTACAATG
                            CTCF (-731/-719)
 -750 TAGCTAAAGC ACAATTATTT CATGCAGTAC CTGACTACTT GAAATATATA TTTTTTAAAC
 -690 AGACTGCTTC AAAATCTCCA TGTAGAACCC GACACAACTA CATCGGTATT CTGTTACTCT
 -630 GCAACTGAAA AAATCATTTC CTTGACAGAT AAAACATAGT TGGACATTCG TGGCAGCTGG
 -570 ATGTATAAAA TATCACAGAC AAAAAGGAGG AGATACTGAC AAGGTGCACT TACAGAGCAG
 -510 TAATCTTAGG AACACACTAC TCCTACTTGT CTCAGTGGGA GACAAGTGGG AGAAGCATGT
 -450 CTCAACTTTG GTATTTTGGA AAAAATGAGA GGCAGCATGT AGTCCTTTTC AAACATTATT
 -390 TTCTCCAGGC TGTTGGCAAC CTCAAATCAC CACAGAAAAG TTTAAGATTC CTCTTAATTA
                                      HNF3α (-300/-292)
 -330 CAGTCTGCCT GTCCACATCA GTGGGAGTTT TGTTGGCACC TTTCAGAAGA TTGCTCATGA
      HNF3α (-269/-262)
 -270 GTTACACTGT GAGACACAGA GGCAAACTAC AGCAGAGAGC GTTCATCCCA ATACACCAGT
 -210 CTTGCTGCAT GGAGCAACCC ACCTCCACAA AATCCTTGCA ACCATCTCTT CCACTTTTGT
 -150 GCTTTGCTAC CTAGAGATTA ACTGCGCATG AGGATCCTTC AAAGAACAGA TCTGTGATTT
                                CDX2 (-65/-57)
  -90 ACCTATCAAG TATTAACGTC ATTTTTACAT TCCAAACCCC ACACGGAAAA TATTACATCT
                                        TSS
  -30 GATTCTCAAG TGCTACTTGG TGGAAAGAAA GGTTAAATGA CTTGCTTGTG ACTCTTTCAC
```

Figure S1. Prediction of transcription factors in the promoter region of chicken *LEAP2*.
